# Nucleic acid liquid biopsies in Alzheimer's disease: current state, challenges, and opportunities

**DOI:** 10.1016/j.heliyon.2022.e09239

**Published:** 2022-04-13

**Authors:** Tabea M. Soelter, Jordan H. Whitlock, Avery S. Williams, Andrew A. Hardigan, Brittany N. Lasseigne

**Affiliations:** a Department of Cell, Developmental and Integrative Biology, The University of Alabama at Birmingham, AL, USA; b Department of Neurosurgery, Duke University Medical Center, Durham, NC, USA

**Keywords:** Liquid biopsy, Circulating biomarkers, Alzheimer's disease, Neurodegeneration, Cell-free, Diagnosis, Nucleic acid

## Abstract

Alzheimer's disease (AD) is the most common neurodegenerative disease and affects persons of all races, ethnic groups, and sexes. The disease is characterized by neuronal loss leading to cognitive decline and memory loss. There is no cure and the effectiveness of existing treatments is limited and depends on the time of diagnosis. The long prodromal period, during which patients' ability to live a normal life is not affected despite neuronal loss, often leads to a delayed diagnosis because it can be mistaken for normal aging of the brain. In order to make a substantial impact on AD patient survival, early diagnosis may provide a greater therapeutic window for future therapies to slow AD-associated neurodegeneration. Current gold standards for disease detection include magnetic resonance imaging and positron emission tomography scans, which visualize amyloid β and phosphorylated tau depositions and aggregates. Liquid biopsies, already an active field of research in precision oncology, are hypothesized to provide early disease detection through minimally or non-invasive sample collection techniques. Liquid biopsies in AD have been studied in cerebrospinal fluid, blood, ocular, oral, and olfactory fluids. However, most of the focus has been on blood and cerebrospinal fluid due to biomarker specificity and sensitivity attributed to the effects of the blood-brain barrier and inter-laboratory variation during sample collection. Many studies have identified amyloid β and phosphorylated tau levels as putative biomarkers, however, advances in next-generation sequencing-based liquid biopsy methods have led to significant interest in identifying nucleic acid species associated with AD from liquid tissues. Differences in cell-free RNAs and DNAs have been described as potential biomarkers for AD and hold the potential to affect disease diagnosis, treatment, and future research avenues.

## 1 Introduction

### 1.1 Background

Neurodegenerative diseases affect millions of patients worldwide, emphasizing the importance of early disease detection to increase the quality of life for patients and their families. Diseases caused by the degeneration of the nervous system portray a variety of phenotypes, including widespread memory loss and overall cognitive decline [1]. Parkinson's disease (PD), Amyotrophic lateral sclerosis (ALS), Alzheimer's disease (AD), Vascular dementia, and Pick's disease are among the most prevalent neurodegenerative diseases, with AD being the most common type affecting persons of all races, ethnic groups, and sexes [1]. There are more than 40 million patients with AD worldwide and they can generally be categorized into early- and late-onset groups, defined by the age of onset of the disease [2]. Patients who develop symptoms before age 65 experience early-onset AD (EOAD), accounting for roughly 5% of all AD cases [3]. Only 10–15% of EOAD patients have an identified inherited autosomal dominant genetic pattern; therefore, the remaining cases are considered sporadic and have an unidentified cause [3]. Interestingly, individuals with identified genetic forms of EOAD often experience symptoms in their 30s and 40s in an extremely aggressive form of the disease compared to the low-profile progression observed in late-onset AD (LOAD) patients [3]. Despite extensive research on AD progression and drug development, no cure or mitigating therapeutic has been approved since 2003, making successful AD treatments a continually unsatisfied demand [4, 5].

Current diagnosis is based on clinical criteria which incorporate information on the patient's history, associated with memory loss and behavioral changes, accumulated through questionnaires of family members and others close to the patient [6]. Brain imaging technologies such as magnetic resonance imaging (MRI) and positron emission tomography (PET), which visualize protein depositions and aggregates in the brains of AD patients, are utilized as supportive measures to increase the certainty of previous clinical diagnoses [6, 7]. Despite being powerful tools and the current gold standard for AD diagnosis and detection, their inability to distinguish between different neurodegenerative disorders and the extreme monetary burden of these methods on patients and the healthcare system are considerable drawbacks [7].

Liquid biopsy, defined by the U.S. National Cancer Institute as the analysis of cell-free circulating tumor DNA and other nucleic acid species released into the blood in cancer, may address the pitfalls of the aforementioned diagnostic standards [8, 9]. Recent advances in liquid biopsy research in oncology have led to their clinical integration in the field of precision oncology, which has opened up the possibility of applying the same diagnostic tools to neurodegenerative diseases such as AD. However, a major hurdle in AD detection is the disease's long prodromal period, which coincides with the preclinical stage of the disorder, during which patients undergo small changes in their cognitive abilities resulting in minor effects on their ability to live a normal life [10]. Molecular diagnostics that allow for earlier diagnosis, evaluation of disease progression, and possible therapeutic monitoring are needed, as current diagnostic methods and interventions are often not employed until later in the disease course, greatly affecting patient outcomes. An alternative approach that adapts liquid biopsy protocols from oncology research may improve the detection of neurodegenerative disorders.

In order to implement liquid biopsy as a detection method in AD, potential biomarkers need to be identified. The brains of AD patients are characterized by disease hallmarks such as neuritic plaques caused by amyloid β aggregation, neurofibrillary tangles composed of hyperphosphorylated tau proteins, and neuroinflammation, which are thought to collectively lead to the development of dementia and cognitive impairment distinctive with AD [11, 12]. These disease hallmarks have been considered as biomarkers for liquid biopsies in AD, as well as various nucleic acid species. Nucleic acids leak from the brain into surrounding tissues such as the cerebrospinal fluid (CSF), which surrounds the brain and spinal cord, and into the bloodstream during aging [13, 14]. While precision oncology commonly uses blood among other tissues for liquid biopsies, determining the most beneficial and least invasive tissue for AD detection is still being elucidated [15].

### 1.2 Challenges

AD's heterogeneous nature is at the foundation of many challenges accompanied with disease detection, biomarker identification, drug development and repurposing, and treatment. Different age phenotypes, genotypes, and etiology lead up to near-identical neuropathology of the disease, impeding the development of early detection methods and possible treatments [16]. Hence, elucidating a universal early disease detection biomarker panel for AD remains challenging. Multiple biofluids have been explored as potential candidates for liquid biopsies in neurodegenerative diseases with regard to collection, storage, and sensitivity. Despite being an ideal candidate for high specificity and sensitivity of AD biomarkers, CSF acquisition from patients is suboptimal. CSF collection methods are invasive and painful. The lumbar puncture, for example, requires the insertion of a large, hollow needle into the subarachnoid space for removal or collection of CSF [7]. Lumbar puncture requires a skilled operator and there are risks to the procedure including possible infection, bleeding and CSF leak with undesirable side effects such as headaches, dizziness, and nausea. Consequently, there would be significant challenges in the adoption of lumbar puncture for widespread diagnosis or screening efforts [17].

These problems can be circumvented by utilizing blood collections, which can be performed repeatedly and are much less invasive. Additionally, aseptic blood collection protocols, processing, and proper temperature storage are commonly performed and are accessible nearly globally [9]. The World Health Organization (WHO) and others provide norms and standards for blood collection with room for further improvement [18, 19, 20]. A drawback of using blood for early disease detection is biomarker specificity and sensitivity, which is affected due to the low permeability of the blood-brain barrier (BBB), affecting biomarkers entering the bloodstream. The BBB is divided into multiple barriers primarily dependent on their location in the brain, all of which are affected by aging [13]. Over 20 research studies have implicated the breakdown of the BBB in samples for AD patients, before the onset of symptoms associated with AD or mild cognitive impairment (MCI), a pre-dementia form of the disease [21]. This potential consequence may aid biomarker detection from blood collections. However, overcoming the BBB is crucial to increase the usefulness of blood for early detection of biomarkers originating in the brain, an avenue that is currently being pursued [22]. While CSF provides greater sensitivity and specificity to the central nervous system (CNS) nucleic acid biomarkers than blood, standardized protocols need to be implemented for biomarker analyses in blood and CSF. Especially CSF collection protocols lack standardization, greatly introducing variation to analyses of biomarkers [23]. In order to employ CSF to its full potential for liquid biopsies, there is a need for universal criteria and unified protocols to prevent inter-laboratory and -study variation [24]. Despite their wide use, blood collection protocols require further adaptation for applications in circulating biomarkers.

A vital issue in AD is the long prodromal period that precedes the onset of noticeable disease symptoms. It facilitates the disease's unimpeded progression due to a lack of early detection and treatment. Early detection would serve primarily as a neuroprotective measure, providing the opportunity to intervene early with the goal of stopping or slowing disease progression and ultimately increasing the patient's quality of life [25, 26]. Repeatable blood collection and disease screening using liquid biomarkers could provide relief to patients who remain undiagnosed until the onset of severe symptoms. While there are no cures for AD, many drugs have been proposed and have undergone clinical trials, but 99.6% of AD clinical trials have failed [25]. A proposed cause for the high failure rate in AD clinical trials is the late initiation of the study. Most studies begin after onset of AD symptoms, which greatly affects outcomes since significant neuronal loss has occurred [25]. Therefore, early detection might also benefit drug trials and clinical studies by targeting the disease before it has progressed too far. However, initial symptoms of MCI, which can lead to AD, are usually misidentified as typical aging, a problem that may be resolved through the implementation of liquid biopsies and their potential for early disease detection, which will lessen false diagnoses [27].

Likewise, as most AD research heavily relies on the use of pre-clinical models, liquid biopsy can have an effect on the development of novel and more accurate models to study disease mechanisms, which are hard to observe in humans. AD mouse models are an alternative tool to study disease progression and have tremendously aided our understanding of the disease. Many models are for EOAD and models accurately mimicking late-onset forms of the disease appear lacking [28]. Therefore, conclusions drawn from early-onset mouse models are often inaccurate in the context of LOAD because EOAD mouse models mimic inherited forms of AD while neglecting to represent sporadic late-onset forms [29]. The underlying mechanisms of mouse neurodegenerative diseases may differ significantly from those in humans despite similar disease progression [30]. This differentiation further implicates the importance of creating predictive mouse models that accurately mirror human AD progression mechanisms. Liquid biopsies hold the potential to uncover underlying LOAD mechanisms at the preclinical stage, giving rise to the possibility to reorganize and reinvent AD mouse models to include late-onset forms of AD instead of being restricted to genetic EOAD.

Finally, technical and sequencing challenges associated with liquid biopsy also remain unaddressed. Next-generation sequencing (NGS) and droplet digital polymerase chain reaction (ddPCR) have previously been utilized to detect circulating tumor DNA from blood with high sensitivity in cancer [31]. ddPCR, an ultrasensitive targeted method, provides fast, inexpensive, and sensitive detection for cancer-specific mutations in liquid biopsy [32]. On the other hand, NGS methods for liquid biomarkers include targeted and untargeted detection approaches, which have the ability to perform massively parallel sequencing [32]. Unfortunately, targeted NGS methods, while showing high sensitivity, are limited by a predefined list of genes. Untargeted NGS methods are not limited to such predefined lists and are able to detect entirely novel changes at the cost of lower sensitivity, increased cost, and the necessity for higher input sample volume — which is particularly detrimental for samples like brain tissue or CSF, which are difficult to acquire [32]. Keeping these limitations in mind, cell-free DNA (cfDNA) detection methods require standardization in order to be introduced into the clinical setting [31, 32]. Sequencing methods for small and long RNA-seq have been shown to create large inter-laboratory variation dependent on respective protocols. A study testing the accuracy and reproducibility of small RNA-seq library preparation methods was able to show decreases in inter-laboratory variation by 100-fold through the implementation of standardized approaches [33]. Therefore, standardization of analytical and post-analytical factors, including experimental design, sequencing protocols, quality control, and raw data processing is necessary to overcome current challenges associated with targeted and untargeted approaches in liquid biomarker research [31, 33]. Despite being used in precision oncology, these methods are broadly applicable to AD, therefore addressing these limitations is a challenge for liquid biopsy studies in AD.

Despite some of the challenges with liquid biopsy development in AD, there remains great promise in their application: early disease detection, therapeutic monitoring, increased understanding of underlying disease mechanisms, and advances in pre-clinical models to include LOAD forms for future studies. These potential findings will advance our understanding of AD etiology, progression, and treatment with the goal of providing relief from the massive health burden caused by AD and increasing patients’ quality of life.

## 2 Main text

### 2.1 Liquid biopsy sources and tissues

Several tissues have been examined as potential candidates for liquid biopsies in neurodegeneration, including AD. CSF has been shown to reflect biochemical changes in the CNS [7]. Despite its useful applications, obtaining CSF is invasive, requiring lumbar puncture resulting in significant discomfort and associated risks, as well as its limited availability hindering regular sample collection for repeated testing [7]. Conversely, blood collection is easily accessible and affordable, allowing for repeated collection with significantly less pain and discomfort. This aids in the development of non-invasive pre-symptomatic screening protocols for AD despite concerns around its lower specificity and sensitivity for CNS biomarkers due to the BBB [7, 34]. Finally, there have been reports of the potential usefulness of ocular, olfactory, and oral fluids for liquid biopsies. Multiple studies have shown differential ocular protein and miRNA levels between AD patients and healthy controls [35, 36]. Koronyo and others highlight the practicality of detecting elevated protein levels of amyloid β in the retina through imaging in 10 AD patients and 6 healthy controls. At the same time, another study identified the elongation factor 4E in tear fluid of over half of the AD samples, which was absent in all controls, as well as an increase in total miRNA quantity in all 9 disease samples compared to 9 MCI samples and 15 age-matched controls [35, 36]. Significant increases in tau and phosphorylated tau protein levels were also detected from olfactory fluids of AD patients, while controls did not exhibit such elevations [37]. Finally, Yilmaz and others described changes in concentration of 22 metabolites in oral fluids of patients with MCI and AD compared to healthy controls [38]. Multiple studies have also shown differences in amyloid β as well as phosphorylated tau concentrations in oral fluids of AD patients and controls, which has been reviewed recently [39]. Notably, most of the studies on oral, olfactory, and ocular fluids for liquid biopsies in AD are focused on protein aggregates such as amyloid β and tau that are outside of the scope of this review, which focuses primarily on nucleic acid species like cell-free RNAs and DNAs. However, expansion of protein-aggregate-focused studies to include nucleic acids will increase the likelihood of oral, olfactory, and ocular fluids to be implemented for liquid biopsies in AD. While being in the preliminary stages of research and showing minimal protein concentrations, the high accessibility and non-invasive nature of these fluids indicate an important future role in at-risk patients and early disease detection during the prodromal period of AD through repeated collections, especially when jointly implemented with other biomarkers or diagnostic approaches [7, 35, 36, 37, 38]. Due to varying levels of accessibility of samples and specificity, studies reporting nucleic acids as potential biomarkers in AD are often limited to blood and CSF collections. Here we report the current state, challenges, and opportunities of nucleic acid liquid biopsy markers across biofluids in AD (Table 1).

**Table 1:** Nucleic Acid Biomarkers across biofluids in AD.

Biomarkers	Description	Nucleic Acid	Source
miR-132 [40,41],miR-212 [40], miR-342-5p [42], miR-200a-3p [43], miR-502-3p [43], miR-142-3p [43], miR-483-5p [43], miR-486-5p [43], miR-30b-5p [43], miR-34a [44], miR-146a [44]	ncRNAs that consist of 20–22 nucleotide sequences and are important for the regulation of gene expression. Studied most in depth as a biomarker for AD	miRNA	Plasma
miRNA-4422 [45], miR-34c [46], miR-193b [47], miR-125b [48,49,50,43], miR-23a [49], miR-26b [49], miR-223 [50], miR22-3p [48,[51], [52], [53], [54]]			Serum
miR-135a [55], miR-146a [48,53,44,56], miRNA-9 [48,46], miR-101 [46], miR-34a [44], miR-29a [44], miR-29b [44]			CSF
miRNA-200b-5p [35]			Tears
tRNA-derived RNA fragments (tRFs) [57, 58]	composed of 73–90 nucleotides and are primarily responsible for translating the messenger RNA (mRNA) sequence into protein	tRNA	-
5′ tRNA halves [59]			Serum
51A [60], BACE1 [54,61,62,60,63,64], BACE1-AS [65]	ncRNAs that consist of sequences of >200 nucleotides and lack open reading frames and protein-coding abilities	lncRNA	Plasma
BACE1-AS [66, 67]			Blood
BACE1-AS [66, 67]			Serum
MALAT1 [68]			CSF
cf-mtDNA [69, 70]	double-stranded fragments of 150–200 base pairs in length	cfDNA	Plasma
cf-mtDNA [71, 72]			CSF

### 2.2 miRNAs

MicroRNAs (miRNAs) are non-coding RNAs (ncRNAs) that consist of 20–22 nucleotide sequences and are important for the regulation of gene expression [73, 74]. Cellular damage, including injury to the brain tissue in AD, can lead to changes in the expression levels of miRNAs in the blood and CSF of patients with neurodegenerative diseases [75]. A major advantage of using miRNAs to detect changes in the biochemical processes of the brain is their ability to travel across the BBB in extracellular vesicles (EVs) [76]. EVs are secreted lipid bilayer vesicles that are subdivided into three types: microvesicles, exosomes, and apoptotic bodies [77]. Their involvement in cell-cell communication has increased their importance in circulating biomarkers for liquid biopsy, as they are able to encapsulate and carry nucleic acid species [77]. As most cell types release EVs and cargo is protected against enzyme degradation, EVs have been investigated for their transport abilities as well as a potential biomarker for AD [78, 79, 80]. Exosomal miRNAs, due to their ability to cross the BBB unharmed, have been a major focus in this field (reviewed in [81]). Some of the latest publications on exosomal miRNAs feature miR-193b, which was found to be significantly elevated in serum of MCI and AD patients (p < 0.05) [47], and miR-135a, showing an increase in the CSF of MCI and AD patients compared to control (p < 0.05) [55]. A recent review highlighted the potential importance of miR-132, which was previously identified in a study of plasma exosomes of AD patients along with miR-212 [40]. However, miR-132 was not able to distinguish MCI and AD patients [41].

Additionally, miRNA molecules are detected in increased abundance and are more stable than higher molecular weight RNA molecules [82]. Serum-derived miRNA stability outperformed large RNA molecule stability in a multitude of conditions, including changes to pH levels, long-term storage, and increased freeze-thaw cycles [83, 84]. Methods for miRNA profiling in liquid tissues include microarray analysis and NGS approaches. However, progress has been limited by low circulating miRNA concentrations for which profiling methods usually require validation by RT-qPCR due to high miRNA variability, specifically in array analyses [82, 85, 86]. Fortunately, TaqMan Low Density array (TLDA) assays, which perform hundreds of simultaneous qPCR reactions and do not require RT-qPCR for further validation [87], are rapid and cost-effective [88] and have been used for miRNA profiling in plasma [42, 89, 90], and CSF [91]. One particular study was able to determine that reduced levels of miR-342-5p in plasma are associated with a faster cognitive decline in a cohort of 19 women [42]. However, it is difficult to precisely identify the tissue of origin of detected miRNAs because amounts of EVs, plasma lipoproteins, and proteins, as well as the type of miRNA extraction method performed, generate expression level biases of miRNAs that hinder tissue of origin identification [75].

Interestingly, miRNAs have been described as the most abundant nucleic acids in CSF and brain-derived extracellular fluid in patients with AD and significant expression upregulation of miRNA-9, miRNA-125b, miRNA-146a, and miRNA-155 has been shown in post-mortem brain tissues of 6 AD and 6 controls through array analyses [48]. On the other hand, these results have not been uniformly validated, especially in miRNA-146a, which has also been described as downregulated in CSF and hippocampus [44, 53], and shows no differences in plasma between AD and controls in another study [92]. Additionally, a study in CSF and serum of AD patients and controls found downregulation of miRNA-9, a finding opposing previous studies, and miRNA-101 in CSF, while miR-34c was elevated [46]. The same study also detected miRNA differences across AD stages. One potential explanation for these varying results is patient age and AD stage. Multiple studies indicate the importance of accounting for disease stage, as different miRNAs were detected at different timepoints in a mouse model of AD [93]. For example, miRNA-146a expression seems to be upregulated in earlier stages and downregulated in later stages of the disease, further emphasizing AD heterogeneity [53]. A study in rats investigated the role of miRNA-146a in inflammation of the brain and suggests its involvement in neuroinflammation after performing a Pearson correlation analysis that indicates its potential role as an inflammation indicator [56]. An array and qRT-PCR study describes downregulation of miR-125b, miR-23a, and miR-26b in 22 AD patients compared to 26 controls in serum and concluded that miR-125b is able to distinguish between AD and non-AD with 82% accuracy [49]. The findings of miR-125b are further supported and extended, as the combination of miR-125b and miR-223 (identified in this study) increased AD prediction sensitivity and specificity than either miRNA by itself [50]. The indication that multiple miRNAs increase the strength of disease prediction in AD is further supported in more recent studies [91, 94]. These findings suggest that the creation and installment of miRNA panels for liquid biopsy in AD may improve its diagnostic strength. Nagaraj and others generated a panel of 6 miRNAs (6miR), comprised of 3 previously identified miRNAs (200a-3p, 502-3p, and 142-3p) and 3 newly identified miRNA candidates (483-5p, 486-5p, and 30b-5p), which was able to successfully separate control and MCI patients from each other in blood plasma [43].

While many different miRNAs have been indicated to be involved in the neuropathology of the disease, it is worth noting that miRNA-9, miRNA-125b, and miRNA-146a contribution has been evaluated repeatedly and shown promising results for AD detection [44, 48, 51, 52, 53, 54]. However, another study indicated the importance of miRNA-4422 on the expression dysregulation of beta-secretase 1 (BACE1) and gamma-secretase activating protein (GSAP) genes, which have been described to influence Aβ42 deposition in AD [45]. A study in tears from AD patients and controls found elevated total miRNA abundance as well as specifically miRNA-200b-5p, a potential fluid biomarker, in AD samples compared to controls [35].

Additionally, many studies aim to develop diagnostic models using cutting-edge computational approaches, including statistical tests comparing age-matched healthy controls to AD samples to discover novel biomarkers. Sheinerman and others used serum samples from patients with various neurodegenerative diseases and age-matched controls to determine that statistical approaches like. NET statistical packages and correlation analyses were able to distinguish healthy from affected individuals as well as to differentiate between neurodegenerative diseases using brain-enriched miRNAs [95]. Furthermore, machine learning approaches successfully differentiated AD patients from controls based on the expression levels of 21 miRNAs (18 of which were significantly correlated with neurodegenerative disease pathology) in blood, further implicating their utility as early diagnostic markers [96]. Another group developed a database of miRNAs differentially expressed in blood-related fluids and CSF of neurodegenerative diseases to recognize miRNA profiles specific to particular neurodegenerative diseases. This global miRNA study describes the intrinsic relationship between miRNAs and neurodegenerative diseases using bioinformatic approaches to identify therapeutic and diagnostic targets of miRNAs in neurodegenerative diseases [97].

Provided that findings describing the potential of miRNAs to detect neurodegenerative diseases, specifically AD (recently reviewed in [98, 99, 100, 101]), are validated further experimentally and computationally, miRNAs have the potential to drive forward the development of early-detection diagnostic tools for AD. Recent efforts from meta-analyses that aim to identify circulating miRNAs for AD detection have and will further increase our understanding of miRNAs as liquid biopsy markers in AD [102, 103, 104]. Therefore, the aforementioned meta-analyses and the propositions of biofluid panels for the detection of AD may allow liquid biopsy establishment in AD diagnosis.

### 2.3 tRNAs

Transfer RNAs (tRNAs) are composed of 73–90 nucleotides and are primarily responsible for translating the messenger RNA (mRNA) sequence into protein [105, 106]. In recent years, tRNA-derived RNA fragments (tRFs) have become a focus in human health and disease studies, and these degradation products have been shown to play regulatory roles in cancer and neurological disorders [57]. Deficiencies in tRNA metabolism and processing enzymes have been linked to neurodegenerative disease, specifically in ALS and PD [107, 108]. Defects in the RNA kinase CLP1 result in impaired pre-tRNA cleavage, increasing oxidative stress on neuronal cells, which drives cell degeneration [109, 110, 111, 112]. Similar to miRNAs, tRNAs and tRFs have been shown to enter circulation and avoid depletion in blood and serum due to their association with circulating extracellular vesicles, thus implicating their utility as a reliable circulating biomarker for AD [58]. Additionally, the presence of tRNA derivatives like stable 5′ tRNA halves has been confirmed in mouse serum despite the derivatives’ lack of connection to extracellular vesicles [59]. Utilizing circulating tRNAs and their derived fragments in neurodegenerative diseases have been largely understudied, even though they exhibit implications linked to neuronal cell death due to oxidative stress. Therefore further studies need to be conducted in this space.

### 2.4 lncRNAs

Long non-coding RNAs (lncRNAs) are ncRNAs that consist of sequences of >200 nucleotides and lack open reading frames and protein-coding abilities [113, 114]. Nonetheless, lncRNAs have been implicated in AD pathology by regulating mRNA stability and have been described repeatedly as differentially expressed in animal models and patients of AD [114]. Early clustering attempts by the GENCODE consortium revealed that about 40% of lncRNAs are highly expressed in the brain, further delineating its potential role in neuropathology and utility as a diagnostic tool for early detection of the disease [115]. Since then, lncRNAs have been found to be dysregulated in brain tissues of AD mouse models compared to healthy and age-matched controls and demonstrate differential expression within disease models in an age-dependent manner [116, 117]. Attempts to characterize lncRNAs involved in disease neuropathology have repeatedly described the involvement of lncRNA BACE1 in AD [61, 62]. BACE1 is a crucial regulator in amyloid β production, a rate-limiting step in AD progression [54]. Interestingly, lncRNA BACE1 levels were highly upregulated in plasma of AD patients when compared to controls in multiple studies, while other lncRNAs did not show such significant alterations [54]. Although BACE1 holds potential as a diagnostic biomarker, some studies have also proposed its use as a therapeutic target through the systematic delivery of BACE1 small interfering RNAs (siRNAs) and its inhibitors [60, 63, 64]. Furthermore, the BACE1 antisense transcript has also been demonstrated to be significantly elevated in the peripheral blood of 30 AD patients compared to their 36 age-matched controls (p-value < 0.01), as well as in serum samples [66, 67]. These findings were expanded by Fotuhi et al. in a study of 45 AD and 36 controls patient plasma samples, which showed no significant difference between AD and controls. After accounting for disease stage and dividing the AD group into pre-AD and full-AD, there were significant differences in the BACE1 antisense transcript between all groups [65]. Studies investigating lncRNAs as potential biomarkers are not limited to BACE1, as PCA3, HOTAIR, MALAT1, and 51A are also investigated, with 51A showing increased levels in AD patient plasma [60]. Lower levels of relative MALAT1 expression in CSF from AD samples compared to controls were also observed [68]. An RNA-seq study identified several lncRNAs that were co-expressed with common AD-related genes [118]. Additionally, another RNA-seq study in ALS also described lncRNA dysregulation, and similar implications in PD, further indicating lncRNA utility in AD and other neurodegenerative diseases [119, 120]. LncRNAs have also been described in brain tissues as well as specific cell types in AD, further indicating their potential role as a nucleic acid in liquid biopsies in AD (Reviewed in [121, 122]). Finally, lncRNAs are also detectable in plasma- and CSF-derived EVs, however, studies are insufficient and require further investigation (Reviewed in [123]). Overall, there is considerable evidence for the involvement of lncRNAs in neurodegenerative diseases, while further research is necessary to fully understand their utility as a disease biomarker.

### 2.5 cfDNAs

Cell-free DNAs (cfDNAs) are double-stranded fragments of 150–200 base pairs in length [124]. Recently, cfDNA has become increasingly important for disease screening and early detection because cfDNA is released into circulation and insinuates the presence of disease [125]. The field of oncology has implemented cfDNA techniques to accurately screen for various cancer types, an avenue less pursued in neurodegeneration [126]. Hence, cfDNA has become of particular interest as a biomarker for neurodegenerative diseases [127, 128]. Neuronal cell death, a process common in AD progression, releases DNA fragments into the bloodstream, which are hypothesized to be useful as a biomarker [127, 129]. A study on MCI indicates that circulating cfDNA holds the potential to be utilized as a biomarker for overall cognitive decline [130]. However, cfDNA levels have been shown to vary significantly between individuals [131]. If the variation is unrelated to the disease state and due to inconsistencies in sample collection, processing, or analysis, this is a potential limitation of this particular nucleic acid. Additionally, cell-free mitochondrial DNA (cf-mtDNA) has been pursued as a potential AD biomarker as well. Detected in both plasma and CSF, damaged mitochondrial DNA is highly stable with low variability, which is particularly useful for consistent analytical approaches to distinguish between AD and non-AD [69]. Multiple studies have shown cf-mtDNA depletion in AD samples as a useful biomarker in neurodegenerative diseases, a phenomenon not observed in healthy controls [69, 70]. Both studies showed low levels of cf-mtDNA in CSF of AD and PD patients and proposed it as an early diagnostic biomarker for neurodegenerative diseases [69, 70, 132]. A recent continuation of this work by Podlesniy and others validated reduced levels of cf-mtDNA in the CSF of AD patients compared to controls, which was decreased by 31% in the AD group [71]. However, an opposing study in post-mortem ventricular CSF across multiple neurodegenerative diseases did not detect significantly lower levels of cf-mtDNA in AD [72]. While the utility of cfDNA in neurodegenerative diseases is not yet well-understood, circulating biomarker research is quickly expanding to include nucleic acids such as cfDNA as an early detection biomarker.

## 3 Conclusions

Liquid biopsy has only recently been accepted as a detection method for AD and other neurodegenerative diseases with significant development due to technical advancements and increased understanding of AD neuropathology. Continued development is underway to expand the implementation and usefulness of the approach. Since most neurodegenerative diseases do not have a cure, the demand for early detection methods is especially high to slow disease progression and increase patients' quality of life. AD drug trials’ sizable failure rates call for changes in the approaches taken to research and treat the disease, a problem for which liquid biopsy can provide relief by uncovering novel therapeutic targets for future trials [9]. As an innovative tool it holds the potential to overcome limitations of traditional methods by improving early disease detection and screening through less-invasive techniques [133]. Additionally, diagnoses using minimally-invasive liquid biopsy approaches will allow for serial collections throughout aging, which resemble annual blood panel screenings in the medical field. This permits early disease detection during the prodromal period of the disease, which currently is often mistaken as normal aging. Therefore, reliable nucleic acid circulating biomarkers like cell-free RNAs and DNAs may address an unmet need and identify AD before its devastating effects on the brain and its associated cognitive decline manifest. This generates a unique opportunity to study and treat pre-symptomatic as well as early AD, disease stages not well understood and lacking treatment at the moment [134].

While the field of oncology has made great strides towards the successful implementation of liquid biopsies, development in neurodegenerative diseases trails behind. Multiple liquids have been proposed for the detection of AD biomarkers and while the use of neural-derived blood extracellular vesicles has been proposed as a reliable and robust method for AD detection and treatment, it has not received the same attention as blood and CSF [26]. Similarly, the usefulness of oral, ocular, and olfactory fluids remains understudied. As the least invasive fluids for detecting AD, these fluids hold ample potential for liquid biopsies but require further studies to determine their true potential, especially in larger cohorts than currently available [135]. CSF and blood are among the most studied and well-understood liquids for AD detection. A major advantage of CSF is high biomarker specificity and sensitivity, but lumbar puncture is still considered an invasive technique, while blood collections are minimally invasive and have been standardized globally, yet specificity and sensitivity are often lower in this fluid. Furthermore, an increasing number of studies demonstrate the affiliation between CSF and blood-based biomarkers and have indicated the power and usefulness of the latter [9].

Although increasing sample size in future liquid biomarker studies will greatly improve our understanding and help prioritize biomarkers for their clinical implementation, there remain concerns about sample management. In order to identify reliable biomarkers and reduce variation between laboratories and studies, cohort-related, assay-related, pre-analytical, and analytical factors must be considered [136]. Specifically, pre-analytical factors, such as circadian rhythm and time of collection, hormones, centrifugation, storage, and collection tubes, can affect liquid biomarker study results [137]. The Geneva AD Biomarker Roadmap Initiative aims to adapt precision oncology liquid biopsy approaches to AD through to implementation of a 5-phase biomarker development framework [138]. In their 2020 update, it is noted that a lot of work has been done in the first 3 phases, but more work remains to be done in phases 4 and 5, which include the assessment of real world performance of liquid biopsy biomarkers in AD [138]. Implementation of standardized protocols for the collection and pre-analytical processing of CSF and blood for liquid biopsies in AD may address inter-laboratory variation and will allow for comparisons between studies. Attempts to develop a comprehensive CSF protocol for circulating factors has been made, but further refining is necessary [24, 139, 140]. Overall, clinical implementation of standardized protocols will stimulate this area of research and allow for serial sample collections which are useful for early disease detection during the long prodromal period of the disease.

The rapidly increasing discovery of potential biomarkers underscores the urgency of developing comprehensive blood- and CSF-based biomarker panels. Numerous nucleic acids like cfDNAs and RNAs have been proposed for AD as well as amyloid β and phosphorylated tau 一 the hallmarks of the disease. Further standardization of liquid collections and downstream analyses will open new avenues for drug development and treatments, ultimately increasing the quality of life and relieving the global health burden of AD and other neurodegenerative diseases.

## Declarations

### Author contribution statement

All authors listed have significantly contributed to the development and the writing of this article.

### Funding statement

This work was supported by NIH R00HG009678 (to Brittany N. Lasseigne, Tabea M. Soelter also supported), the UAB Lasseigne Lab Start-Up funds (to Brittany N. Lasseigne), and the UAB Blazer Fellowship, UAB Graduate School, United States (to Tabea M. Soelter, Jordan H. Whitlock).

### Data availability statement

No data was used for the research described in the article.

### Declaration of interests statement

The authors declare no conflict of interest.

### Additional information

No additional information is available for this paper.
